# A Step-by-Step Process on Sample Size Determination for Medical Research

**DOI:** 10.21315/mjms2021.28.2.2

**Published:** 2021-04-21

**Authors:** Mohamad Adam Bujang

**Affiliations:** Clinical Research Centre, Sarawak General Hospital, Kuching, Sarawak, Ministry of Health Malaysia, Malaysia

**Keywords:** methods, research, sample size, statistics

## Abstract

Determination of a minimum sample size required for a study is a major consideration which all researchers are confronted with at the early stage of developing a research protocol. This is because the researcher will need to have a sound prerequisite knowledge of inferential statistics in order to enable him/her to acquire a thorough understanding of the overall concept of a minimum sample size requirement and its estimation. Besides type I error and power of the study, some estimates for effect sizes will also need to be determined in the process to calculate or estimate the sample size. The appropriateness in calculating or estimating the sample size will enable the researchers to better plan their study especially pertaining to recruitment of subjects. To facilitate a researcher in estimating the appropriate sample size for their study, this article provides some recommendations for researchers on how to determine the appropriate sample size for their studies. In addition, several issues related to sample size determination were also discussed.

## Introduction

Sample size calculation or estimation is an important consideration which necessitate all researchers to pay close attention to when planning a study, which has also become a compulsory consideration for all experimental studies (1). Moreover, nowadays, the selection of an appropriate sample size is also drawing much attention from researchers who are involved in observational studies when they are developing research proposals as this is now one of the factors that provides a valid justification for the application of a research grant (2). Sample size must be estimated before a study is conducted because the number of subjects to be recruited for a study will definitely have a bearing on the availability of vital resources such as manpower, time and financial allocation for the study. Nevertheless, a thorough understanding of the need to estimate or calculate an appropriate sample size for a study is crucial for a researcher to appreciate the effort expended in it.

Ideally, one can determine the parameter of a variable from a population through a census study. A census study recruits each and every subject in a population and an analysis is conducted to determine the parameter or in other words, the true value of a specific variable will be calculated in a targeted population. This approach of analysis is known as descriptive analysis. On the other hand, the estimate that is derived from a sample study is termed as a ‘statistic’ because it analyses sample data and subsequently makes inferences and conclusions from the results. This approach of analysis is known as inferential analysis, which is also the most preferred approach in research because drawing a conclusion from the sample data is much easier than performing a census study, due to various constraints especially in terms of cost, time and manpower.

In a census study, the accuracy of the parameters cannot be disputed because the parameters are derived from all subjects in the population. However, when statistics are derived from a sample, it is possible for readers to query to what extent these statistics are representative of the true values in the population. Thus, researchers will need to provide an additional piece of evidence besides the statistics, which is the *P*-value. The statistical significance or usually termed as ‘*P*-value less than 0.05’, and it shall stand as an evidence or justification that the statistics derived from the sample can be inferred to the larger population. Some scholars may argue over the utility and versatility of *P*-value but it is nevertheless still applicable and acceptable until now (3–5).

### Why It is Necessary to Perform a Sample Size Calculation or Estimation?

In order for the analysis to be conducted for addressing a specific objective of a study to be able to generate a statistically-significant result, a particular study must be conducted using a sufficiently large sample size that can detect the target effect sizes with an acceptable margin of error. In brief, a sample size is determined by three elements: i) type I error (alpha); ii) power of the study (1-type II error) and iii) effect size. A proper understanding of the concept of type I error and type II error will require a lengthy discussion. The prerequisite knowledge of statistical inference, probability and distribution function is also required to understand the overall concept (6–7). However, in sample size calculation, the values of both type I and type II errors are usually fixed. Type I error is usually fixed at 0.05 and sometimes 0.01 or 0.10, depending on the researcher. Meanwhile, power is usually set at 80% or 90% indicating 20% or 10% type II error, respectively. Hence, the only one factor that remains unspecified in the calculation of a sample size is the effect size of a study.

Effect size measures the ‘magnitude of effect’ of a test and it is independent of influences by the sample size (8). In other words, effect size measures the real effect of a test irrespective of its sample size. With reference to statistical tests, it is an expected parameter of a particular association (or correlation or relationship) with other tests in a targeted population. In a real setting, the parameter of a variable in a targeted population is usually unknown and therefore a study will be conducted to test and confirm these effect sizes. However, for the purpose of sample size calculation, it is still necessary to estimate the target effect sizes. By the same token, Cohen (9) presented in his article that a larger sample size is necessary to estimate small effect sizes and vice versa.

The main advantage of estimating the minimum sample size required is for planning purposes. For example, if the minimum sample size required for a particular study is estimated to be 300 subjects and a researcher already knows that he/she can only recruit 15 subjects in a month from a single centre. Thus, the researchers will need at least 20 months for data collection if there is only one study site. If the plan for data collection period is shorter than 20 months, then the researchers may consider to recruit subjects in more than one centre. In case where the researchers will not be able to recruit 300 subjects within the planned data collection period, the researchers may need to revisit the study objective or plan for a totally different study instead. If the researcher still wishes to pursue the study but is unable to meet the minimum required sample size; then it is likely that the study may not be able to reach a valid conclusion at the end, which will result in a waste of resources because it does not add any scientific contributions.

### How to Calculate or Estimate Sample Size?

Sample size calculation serves two important functions. First, it aims to estimate a minimum sample size that can be sufficient for achieving a target level of accuracy in an estimate for a specific population parameter. In this instance, the researcher aims to produce an estimate that is expected to be equally accurate as an actual parameter in the target population. Second, it also aims to determine the level of statistical significance (i.e. *P*-value < 0.05) attained by these desired effect sizes. In other words, a researcher aims to infer the statistics derived from the sample to that of the larger population. In this case, a specific statistical test will be applied and the *P*-value will be calculated by using the statistical test (which will determine the level of statistical significance).

For univariate statistical test such as independent sample *t*-test or Pearson’s chi-square test, these sample size calculations can be done manually using a rather simple formula. However, the manual calculation can still be difficult for researchers who are non-statisticians. Various sample size software have now been introduced which make these sample size calculation easier. Nevertheless, a researcher may still experience some difficulty in using the software if he/she is not familiar with the concept of sample size calculation and the statistical tests. Therefore, various scholars have expended some effort to assist the researchers in the determination of sample sizes for various purposes by publishing sample size tables for various statistical tests (10–12). These sample sizes tables can be used to estimate the minimum sample size that is required for a study. Although such tables may have only a limited capacity for the selection of various effect sizes, and their corresponding sample size requirements; it is nonetheless much more practical and easier to use.

For some study objectives, it is often much easier to estimate the sample size based on a rule-of-thumb instead of manual calculation or sample size software. Taking an example of an objective of a study that needs to be answered using multivariate analysis, the estimation of an association between a set of predictors and an outcome can be very complicated if it involves many independent variables. In addition, the actual ‘effect size’ can range from low to high, which renders it even more difficult to be estimated. Therefore, it is recommended to adopt the conventional rule-of-thumb for estimating these sample sizes in these circumstances. Although some scholars have initially thought that the concept of rule-of-thumb may not be as scientifically robust when compared to actual calculations, it is still considered to be an acceptable approach (13–15). Table 1 illustrates some published articles for various sample size determinations for descriptive studies and statistical tests.

**Figure 1:**
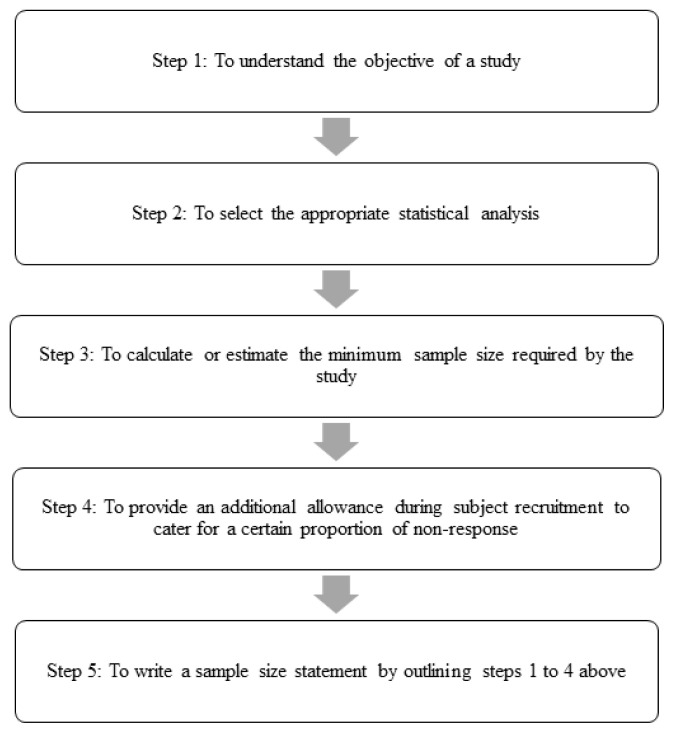
Recommended steps in sample size determination

In brief, the present paper will be proposing five main steps for sample size determination as shown in Figure 1. The following provides an initial description and then a discussion of each of these five steps:

#### Step 1: To Understand the Objective of the Study

The objective of a study has to be measurable or in other words, can be determined by using statistical analysis. Sometimes, a single study may have several objectives. One of the common approaches to achieve this is to estimate the sample size required for every single objective and then the minimum required sample size for the study will be selected to be the highest number of all sample sizes calculated. However, this paper recommends that the minimum sample size be calculated only for the primary objective, which will remain valid as long as the primary objective is more important than all the other objectives. This also means that the calculation of minimum sample size for any other objectives (apart from the primary objective) will only be considered unless they are considered to be equally important as the primary objective. For the development of a research proposal, different institutions may apply different approaches for sample size determinations and hence, it is mandatory to adhere to their specific requirements for sample size determinations.

However, the estimation or calculation of sample size for every study objective can be further complicated by the fact that some of the secondary objectives may require a larger sample size than the primary objective. If the recruitment of a larger number of subjects is not an issue, then it will always be viable to obtain a larger sample size in order to accommodate the sample size requirements for each and every objective of the study. Otherwise, it may be advisable for a researcher to forgo some of the secondary objectives so that they will not be too burdensome for the him/her.

#### Step 2: To Select the Appropriate Statistical Analysis

Researchers have to decide the appropriate analysis or statistical test to be used to answer the study objective; regardless of whether the aim is to determine a single mean, or a prevalence, or correlation, or association, just to name a few. The formula that will be used to estimate or calculate the sample size will be the same as the formula for performing the statistical test that will be used to answer the objective of study. For example, if an independent sample *t*-test has to be used for analysis, then its sample size formula should be based on an independent sample *t*-test. Hence, there is no a single formula for sample size calculation or estimation which can apply universally to all situations and circumstances.

#### Step 3: To Calculate or Estimate the Sample Size

Estimating or calculating the sample size can be done either by using manual calculation, sample size software, sample size tables from scientific published articles, or by adopting various acceptable rule-of-thumbs. Since both the type I and type II errors are already pre-specified and fixed, hence only the effect size remains to be specified in order for the determination of an appropriate sample size. To illustrate this point, it will be easier to demonstrate by using a case scenario as an example. Say a researcher would like to study an effectiveness of a new diet programme to reduce weight. The researcher believes the new diet programme is better than the conventional diet programme. It was found that the conventional diet programme can reduce on average 1 kg in 1 month. How many subjects are required to prove that the new diet programme is better than the conventional diet programme?

Based on Step 1 and Step 2, a researcher has decided to apply the independent sample *t*-test to answer the objective of study. Next, the researcher will need to specify the effect size after having both type I error and power set at 0.05% and 80%, respectively (type II error = 20%). What margin of effect size will be appropriate? This shall depend on the condition itself or the underlying research rationale which can then be further classified into two categories. In the first category, the research rationale is to prove that the new diet programme (for reducing weight) is superior to the conventional diet programme. In this case, the researcher should aim for sizeably large effect size. In other words, the difference between means of the weight reduction (which constitutes part of the effect size for independent sample *t*-test) should be sufficiently large to demonstrate the superiority of the new diet programme over the conventional diet programme.

In the second category, the research rationale is to measure accurately the effectiveness of the new diet programme to reduce weight in comparison with conventional diet programme, irrespective of whether the difference between both programmes is large or small. In this situation, the difference does not matter since the researcher aims to measure an exact difference between them, which means that it can only tolerate a very low margin of difference. In this circumstance, the researcher will therefore only be able to accept the smaller effect sizes. The estimate of effect sizes in this instance can be reviewed either from literatures, pilot study, historical data and rarely by using an educated guess.

The acceptable or desirable effect size that can be found from the literature can vary over a wide range. Thus, one of the better options is to seek for the relevant information from published articles of recent studies (within 5 years) that applied almost similar research design such as used the same treatments and had reported about similar patient characteristics. If none of these published articles can provide a rough estimate of the desired effect size, then the researcher may have to consider conducting a pilot study to obtain a rough estimate of the closest approximation to the actual desired effect size. Besides, historical data or secondary data can also be used to estimate the desired effect size, provided that the researcher has access to the secondary data of the two diet programmes. However, it must be emphasised that deriving the effect size from secondary data may not always be feasible since the performance of the new intervention may still not yet have been assessed.

The last option is to estimate the desired effect size based on a scientifically or a clinically meaningful effect. This means the researcher, through his or her own knowledge and experience, is able to determine an expectation of the difference in effect, and then to set a target difference (namely, effect size) to be achieved. For example, a researcher makes an educated guess about the new diet programme, and requires it to achieve a minimum difference of 3 kg in weight reduction per month in order for it to demonstrate superiority over the conventional diet programme. Although it is always feasible to set a large effect size especially if the new diet programme has proven to be a more rigorous intervention and probably also costlier; however, there is also a risk for the study to might have possibly failed to report a statistically significant result if it has subsequently been found that the actual effect size is much smaller than that adopted by the study, after the analysis has been completed. Therefore, it is usually quite a challenging task to estimate an accurate effect size since the exact value of the effect size is not known until the study is completed. However, the researcher will still have to set the value of effect size for the purpose of sample size calculation or estimation.

Next is to calculate or estimate sample size either based on manual calculation, sample size software, sample size tables or by adopting a conventional rule of thumb. Referring to the example for illustration purposes, the sample size calculation was calculated by using the sample size software as follows; with a study setting of equal sample size for both groups, the mean reduction is set at only 1 kg with within group standard deviation estimated at 0.8 (derived from literature, pilot study or based on a reliable source), type I error at 0.05 and 80% power, a minimum sample size of 11 subjects are required for each group (both for new diet programme and conventional diet programme). The sample size was calculated using Power and Sample Size (PS) software (by William D Dupont and W Dale Plummer, Jr. is licensed under a Creative Commons Attribution-NonCommercial- NoDerivs 3.0 United States License).

#### Step 4: To Provide an Additional Allowance During Subject Recruitment to Cater for a Certain Proportion of Non-Response

After the minimum required sample size has been identified, it is necessary to provide additional allowances to cater for potential non-response subjects. A minimum required sample size simply means the minimum number of subjects a study must have after recruitment is completed. Thus, researchers must ideally be able to recruit subjects at least beyond the minimum required sample size. To avoid underestimation of sample size, researchers will need to anticipate the problem of non-response and then to make up for it by recruiting more subjects on top of the minimum sample size, usually by 20% to 30%. If, for example, the researcher is expecting a high non-response rate in a self-administered survey, then he/she should provide an allowance for it by adding more than 30% such as 40% to 50%. The occurrence of non-response could also happen in various other scenarios such as dropping out or loss to follow-up in a cohort study and experimental studies. Besides that, missing data or loss of records are also potential problems that can result in attrition in observational studies.

Referring to previous example as an illustration, by adding 20% of non-response rate in each group, 14 subjects are required in each group. The calculation should be done as follow:

11/0.8=13.75≈14 subjects.

Likewise, for a 30% non-response rate, the sample size required in each group will then be increased to 16 subjects (11/0.7 = 15.7 ≈ 16).

#### Step 5: To Write a Sample Size Statement

The sample size statement is important and it is usually included in the protocol or manuscript. In the existing research literatures, the sample size statement is written in various styles. This paper recommends for the sample size statement to start by reminding the readers or reviewers about the main objective of study. Hence, this paper recommends all the elements from Step 1 until Step 4 (study objective, appropriate statistical analysis, sample size estimation/calculation and non-response rate) should be fully stated in the sample size statement. Therefore, a proposed outline of this sample size statement of the previous example for two weight-losing diet programmes is as follows:

“This study hypothesised that the new diet programme is better than conventional diet programme in terms of weight reduction at a 1-month follow-up. Therefore, the sample size formula is derived from the independent sample *t*-test. Based on the results of a previous study (cite the appropriate reference), all the response within each subject group are assumed to be normally distributed with a within-group standard deviation (SD) of 0.80 kg. If the true mean difference of the new diet programme versus the conventional diet programme is estimated at 1.0 kg, the study will need to recruit 11 subjects in each group to be able to reject the null hypothesis that the population means of the new diet programme and conventional diet programme are found to be equal with a type I error of 0.05 and with at least 80% power of this study. By providing an additional allowance of 20% in sample recruitment due to possible non-response rate, the required sample size has been increased to 14 subjects in each group. The formula of sample size calculation is based on a study reported by Dupont and Plummer (31).”

## Discussion on Effect Size Planning

Sample size is just an estimate indicating a minimum required number of sample data that is necessary to be collected to derive an accurate estimate for the target population or to obtain statistically significant results based on the desired effect sizes. In order to calculate or estimate sample size, researchers will need to provide some initial estimates for effect sizes. It is usually quite challenging to provide a reasonably accurate value of the effect size because the exact values of these effect sizes are usually not known and can only be derived from the study after the analysis is completed. Hence, the discrepancies of the effect sizes are commonly expected where the researchers will usually either overestimate or underestimate them.

A major problem often arises when the researchers overestimate the effect sizes during sample size estimation, which can lead to a failure of a study to detect a statistically significant result. To avoid such a problem, the researchers are encouraged to recruit more subjects than the minimum required sample size of the study. By referring to the same example previously (new diet programme versus conventional diet programme), if the required sample size is 11 subjects in each group, then researchers may consider recruiting more than 11 subjects such as 18 to 20 subjects in each group. This is possible if the researchers have the capability in terms of manpower and research grant to recruit more subjects and also if there are adequate number of subjects available to be recruited.

After the study is completed, if the difference in mean reduction was found not at least 1 kg after 1 month, then the result might not be statistically significant (depending on the actual value of the within-group SD) for a sample size of 11 subjects in each group. However, if the researchers had recruited 18 subjects in each group, the study will still obtain significant results even though the difference of mean reduction was 0.8 kg (if the within-group SD is estimated to be 0.8, and an equal sample size is planned for both groups, with type I error set at 0.05 and power of at least 80%). In this situation, researcher would still be able to draw a conclusion that the difference in mean reduction after one month was 0.8 kg, and this result was statistically significant. Such a conclusion is perhaps more meaningful than stating a non-significant result (*P* > 0.05) for another study with only 11 subjects in each group.

However, it is necessary to always bear in mind that obtaining a larger sample size merely to show that *P*-value is less than 0.05 is not the right thing to do and it can also result in a waste of resources. Hence, the purpose of increasing the size of the sample from 11 to 18 per group is not merely for obtaining a *P*-value of less than 0.05; but more importantly, it is now able to draw a valid and clinically-significant conclusion from the smallest acceptable value of the effect size. In other words, the researcher is now able to tolerate a smaller effect size by stating that the difference in mean reduction of 0.8 kg is also considered to be a sizeable effect size because it is clinically significant. However, if the researcher insists that the difference in mean reduction should be at least 1.0 kg, then it will be necessary to maintain a minimum sample of only 11 subjects per group. It is now clear that such a subjective variation in the overall consideration of the magnitude of this effect size sometimes depends on the effectiveness and the cost of the new diet programme and hence, this will always require some degree of clinical judgement.

The concept of setting a desired value of the effect size is almost identical for all types of statistical test. The above example is only describing an analysis using the independent sample *t*-test. Since each statistical test may require a different effect size in its calculation or estimation of the sample size; thus, it is necessary for the researchers to be familiarised with each of these statistical tests in order to be able to set the desired values of the effect sizes for the study. In addition, further assistance may be sought from statisticians or biostatisticians for the determination of an adequate minimum sample size required for these studies.

### Another Example of Sample Size Estimation Using General Rule of Thumb

Say a study aims to determine the association of factors with optimal HbA1c level as determined by its cut-off point of < 6.5% among patients with type 2 diabetes mellitus (T2DM). Previous study had already estimated that several significant factors were identified, and then included as three to four variables in the final model consisting of parameters that were selected from demographic profile of patients and clinical parameters (cite the appropriate reference). Now, the question is: How many T2DM patients should the study recruit in order to answer the study objective?

#### Step 1: To Understand the Objective of Study

The study aims to determine a set of independent variables that show a significant association with optimal HbA1c level (as determined by its cut-off point of < 6.5%) among T2DM patients.

#### Step 2: To Decide the Appropriate Statistical Analysis

In this example, the outcome variable is in the categorical and binary form, such as HbA1c level of < 6.5% versus ≥ 6.5%. On the other hand, there are about 3 to 4 independent variables, which can be expressed in both the categorical and numerical form. Therefore, an appropriate statistical analysis shall be logistic regression.

#### Step 3: To Estimate or Calculate the Sample Size Required

Since this study will require a multivariate regression analysis, thus it is recommended to estimate sample size based on the general rule of thumb. This is because the calculation of sample size for a multivariate regression analysis can be very complicated as the analysis will involve many variables and effect sizes. There are several general rules of thumb available for estimating the sample size for multivariate logistic regression. One of the latest rule of thumb is proposed by Bujang et al. (44). Two approaches are introduced here, namely: i) sample size estimation based on concept of event per variable (EPV) and ii) sample size estimation based on a simple formula.

i) Sample size estimation based on a concept EPV 50

For EPV 50, the researcher will need to know the prevalence of the ‘good’ outcome category and the number of subjects in the ‘good’ outcome category to fit the rule of EPV 50 (14, 44). Say, the prevalence of ‘good’ outcome category is reported at 70% (cite the appropriate reference). Then, with a total of four independent variables, the minimum sample size required in the ‘poor’ outcome category will be at least 200 subjects in order to fulfil the condition for EPV 50 (i.e. 200/4 = 50). On the other hand, by estimating the prevalence of ‘good’ outcome at 70.0%, this study will therefore need to recruit at least 290 subjects in order to ensure that a minimum 200 subjects will be obtained in the ‘poor’ outcome category (70/100 x 290 = 203, and 203 > 200).

ii) Sample size estimation based on a formula of *n* = 100 + *50i* (where *i* represents number of independent variable in the final model)

When using this formula, the researcher will first need to set the total number of independent variables in the final model (44). As stated in the example, the total number of independent variables were estimated to be about three to four (cite the appropriate reference). Then, with a total of four independent variables, the minimum required sample size will be 300 patients [(i.e. 100 + 50 (4) = 300].

#### Step 4: To Provide Additional Allowance for a Certain Proportion of Non-Response Rate

In order to make up for a rough estimate of 20.0% of non-response rate, the minimum sample size requirement is calculated to be 254 patients (i.e. 203/0.8) by estimating the sample size based on the EPV 50, and is calculated to be 375 patients (i.e. 300/0.8) by estimating the sample size based on the formula *n* = 100 + 50*i.*

#### Step 5: To Write a Sample Size Statement

There were previously two approaches that were introduced to estimate sample size for logistic regression. Say, if the researcher chooses to apply the formula *n* = 100 + 50*i.* Therefore, the sample size statement will be written as follows:

“The main objective of this study is to determine the association of factors with optimal HbA1c level as determined by its cut-off point of < 6.5% among patients with type 2 diabetes mellitus (T2DM). The sample size estimation is derived from the general rule of thumb for logistic regression proposed by Bujang et al. (44), which had established a simple guideline of sample size determination for logistic regression. In this study, Bujang et al. (44) suggested to calculate the sample size by basing on a formula *n* = 100 + 50*i.* The estimated total number of independent variables was about three to four (cite the appropriate reference). Thus, with a total of four independent variables, the minimum required sample size will be 300 patients (i.e. 100 + 50 (4) = 300). By providing an additional allowance to cater for a possible dropout rate of 20%, this study will therefore need at least a sample size of 300/0.8 = 375 patients.”

## Other Issues

Previously, there are four different approaches to estimate an effect size such as: i) by deriving it from the literature; ii) by using historical data or secondary data to estimate it; iii) by determining the clinical meaningful effect and last but not least and iv) by deriving it from the results of a pilot study. It is a controversial practice to estimate the effect size from a pilot study because it may not be accurate since the effect size has been derived from a small sample provided by a pilot study (52–55). In reality, many researchers often encounter great difficulties in the estimation of sample size either i) when the required effect size is not reported by the existing literature; or ii) if some new, innovative research proposals which may pose pioneering research questions that have never been addressed; or iii) if the research is examining a new intervention or exploring a new research area in where no similar studies have previously been conducted. Although there are many concerns about validity of using pilot studies for power calculation, further research is still being conducted in pilot studies in order to apply more scientifically robust approaches for using pilot studies in gathering preliminary support for subsequent research. For example, there are now many published studies regarding guidelines for estimating sample size requirements in pilot studies (54–61).

## Conclusion

This article has sought to provide a brief but clear guidance on how to determine the minimum sample size requirements for all researchers. Sample size calculation can be a difficult task, especially for the junior researcher. However, the availability of sample size software, and sample size tables for sample size determination based on various statistical tests, and several recommended rules of thumb which can be helpful for guiding the researchers in the determination of an adequate sample size for their studies. For the sake of brevity and convenience, this paper hereby proposes a useful checklist that is presented in Table 2, which aims to guide and assist all researchers to determine an adequate sample size for their studies.

## Figures and Tables

**Table 1:** Summary of published articles related to sample size determination for various statistical tests

		Published articles
a.	To estimate parameters for population	Krejcie and Morgan (10), Lachin (16), Campbell et al. (17), Bartlett et al. (18), Israel (19), Naing et al. (20).
b.	To infer the results for larger population	
	Correlation	Cohen (9), Algina and Olejnik (21), Bujang and Nurakmal (22).
	Intra-class correlation	Fleiss and Cohen (23), Bonett (24), Zou (25), Bujang and Baharum (26).
	Kappa agreement test	Cicchetti (27), Flack et al. (28), Cantor (29), Sim and Wright (30), Bujang and Baharum (11).
	Independent sample *t*-test and paired *t*-test	Lachin (16), Cohen (9), Dupont and Plummer (31).
	One-way ANOVA	Cohen (9), Jan and Shieh (32).
	Pearson’s chi-square	Lachin (16), Cohen (9), Dupont and Plummer (31).
	Cronbach’s alpha	Bonett (33), Bonett (34), Bonett and Wright (35), Bujang et al. (36).
	Sensitivity and specificity	Buderer (37), Malhotra and Indrayan (38), Bujang and Adnan (12).
	Linear regression or Multiple linear regression	Cohen (9), Dupont and Plummer (31), Hsieh et al. (39), Knofczynski and Mundfrom (40), Tabachnick and Fidell (41), Bujang et al. (42).
	Analysis of covariance	Borm et al. (43), Bujang et al. (44).
	Logistic regression	Peduzzi et al. (14), Hsieh et al. (39), Bujang et al. (44).
	Survival analysis	Lachin (16), Lachin and Foulkes (45), Dupont and Plummer (31).
	Cox regression	Peduzzi et al. (13), Hsieh and Lavori (46), Schmoor et al. (47).
	Exploratory factor analysis	Barrett and Kline (48), Osborne and Costello (49), Bujang et al. (50), Bujang et al. (51).

**Table 2:** A step-by-step guide for sample size determination

Steps	Processes	Checklist
Step 1	To understand the objective of study	
	a. The objective of study can be addressed by statistical analysis.	( )
Step 2	To decide the appropriate statistical analysis	
	a. The appropriate statistical test to answer the objective of study has been selected.	( )
Step 3	To estimate or calculate the sample size	
	a. It is necessary to ensure that the basis for which the determination of the effect sizes and/or conditions and assumptions for the use of a rule of thumb are robust and appropriate.	( )
	b. It is necessary to state clearly the planned effect sizes for the statistical test/ the conditions and assumptions for the use of a rule of thumb for sample size estimation.	( )
	c. Sample size is estimated by either i) using a manual calculation; ii) using a sample size software; iii) referring to a sample size table or iv) using a wellrecognised rule of thumb.	( )
	d. It is necessary to ensure that the estimated sample size is feasible to be recruited within the allocated time period for recruitment.	( )
Step 4	To provide additional allowance to cater for the possibility of non-response rate	
	a. It is necessary to decide whether the total non-response rate is acceptable (or not).	( )
	b. It is necessary to adjust the estimated sample size by incorporating an additional allowance to cater for a certain percentage of non-response rate.	( )
Step 5	To write a sample size statement	
	The sample size statement should include the following details:	
	a. The study objective or its hypothesis.	( )
	b. The choice of the statistical test to address the study objective.	( )
	c. It is necessary to state clearly the effect sizes for the statistical test/the conditions and assumptions for the use of a rule of thumb for sample size estimation.	( )
	d. It is necessary to cite all relevant reference(s) or justification(s) supporting the planned effect sizes/condition(s) and assumption(s) for the use of a rule of thumb for sample size estimation.	( )
	e. It is necessary to state clearly the cut-off values for type I error and power, except when the sample size estimation is based on a rule of thumb (then it will become unnecessary to do so).	( )
	f. It is necessary to state clearly the possibility of non-response rate, and to provide an additional allowance to cater for it by recruiting more than the minimum sample size.	( )
	g. To state the sample size to be recruited.	( )
